# Obituary

**Published:** 1905-09-15

**Authors:** 


### OBITUARY.

Dr. William Taft died at Brewster, N. Y., July 23rd, after a short illness of diabetic coma. Dr. William Taft was the oldest son of professor J. Taft, and was a well-known practitioner of dentistry in Cincinnati for many years. He was born July 12th, 1844, and was 61 years old at the time of his death. Dr. Taft graduated from the Ohio College of Dental Surgery in 1863, and practiced in Cincinnati until 1901, when he retired from practice and moved with his family to Brewster, N. Y., where he lived on a farm. He leaves a wife and four sons. The oldest son, J. R. Taft, practices dentistry in Jackson, Mich., and the other sons live at Brewster. Dr. Taft, during his active professional career was considered one of the most expert operators in the West. He was also an expert prosthodontist, his continuous gum work was highly artistic. He was not, like his father, a public professional man, but he was a great reader and kept well posted on all professional matters, and was an occasional contributor to dental literature. He was a graduate of the Ohio Medical College, but never practiced medicine. He was of an artistic temperament and loved music and spent much time with musical and other artistic people. He enjoyed his home and had a genial disposition and loved his intimate friends. He was a great friend and admirer of Dr. H. J. McKellops and Dr. Corydon Palmer, two men who influenced his professional ideas to a large extent. During the last five or six years of his life he came so much under the influence of the disease which caused his death that he was unable to attend to the practice of his profession. He had many friends in the profession who will be sorry when they learn of his death. He made a record as a practitioner of the highest skill and at one time had one of the best practices in Cincinnati. He was buried in Brewster, N. Y.

---

Dr. A. C. McFall, of Mayfield, Ky., died at his home March 28th, 1905. Dr. McFall was a graduate of Washington University Dental Department, of St. Louis, in 1874. He was a well-known and highly respected practitioner of southwestern Kentucky.

